# Seed Priming with Polyethylene Glycol Induces Physiological Changes in Sorghum (*Sorghum bicolor L*. Moench) Seedlings under Suboptimal Soil Moisture Environments

**DOI:** 10.1371/journal.pone.0140620

**Published:** 2015-10-15

**Authors:** Fei Zhang, Jialin Yu, Christopher R. Johnston, Yanqiu Wang, Kai Zhu, Feng Lu, Zhipeng Zhang, Jianqiu Zou

**Affiliations:** 1 Department of Innovation Center, Liaoning Academy of Agricultural Sciences, Shenyang, Liaoning, China; 2 Department of Crop and Soil Sciences, University of Georgia, Griffin, Georgia, United States; Henan Agricultural Univerisity, CHINA

## Abstract

Osmopriming with PEG has potential to improve seed germination, seedling emergence, and establishment, especially under stress conditions. This research investigated germination performance, seedling establishment, and effects of osmopriming with PEG on physiology in sorghum seedlings and their association with post-priming stress tolerance under various soil moisture stress conditions. Results showed that seed priming increased the environmental range suitable for sorghum germination and has potential to provide more uniform and synchronous emergence. Physiologically, seed priming strengthened the antioxidant activities of APX, CAT, POD, and SOD, as well as compatible solutes including free amino acid, reducing sugar, proline, soluble sugar, and soluble protein contents. As a result, seed priming reduced lipid peroxidation and stabilized the cell membrane, resulting in increased stress tolerance under drought or excessive soil moisture environments. Overall, results suggested that seed priming with PEG was effective in improving seed germination and seedling establishment of sorghum under adverse soil moisture conditions. Osmopriming effectively strengthened the antioxidant system and increased osmotic adjustment, likely resulting in increased stress tolerance.

## Introduction

Water stress is a serious agronomic problem worldwide and is one of the most important factors reducing crop productivity. Water stress may arise as a result of excessive soil moisture stress (EM) or water deficit [1]. For sorghum (*Sorghum bicolor* L. Moench) grown in Northern China, water resources are often limited, with drought stress occurring often during the early growing season in spring. In the wet summer season, however, sorghum may suffer intermittent or long-term EM due to excessive rainfall, storm, or flooding. Further, the variation of precipitation in recent years as a result of global warming and climate change often cause fluctuations in soil moisture [2], which may adversely affect sorghum productivity.

In plant cells, increased production of reactive oxygen species (ROS) including hydrogen peroxide (H_2_O_2_), superoxide radicals (O^2−^), singlet oxygen (-O_2_), and hydroxyl radicals (-OH) have been noted to occur during drought [1] and EM [3, 4]. These reactive oxygen species react with cellular constituents, causing a cascade of oxidative reactions including lipid peroxidation, chlorophyll, protein, and pigment degradation, and DNA strand breakage [1, 5–7].

Plants have developed a complex antioxidant system to alleviate the adverse effects caused by ROS. The main free radical scavengers of this antioxidant system include ascorbate, carotenoids, tocopherols, and glutathione, as well as enzymes such as ascorbate peroxidase (APX), catalase (CAT), peroxidase (POD), and superoxide dismutase (SOD)[4, 7, 8]. Antioxidants can react enzymatically and chemically with ROS to remove them from plant cells [1, 4, 7]. For instance, singlet oxygen is quenched primarily by carotenoids [9]. Superoxide radical is transformed to molecular oxygen and hydrogen peroxide by SOD[1].

Plants also cope with water stress by a process known as osmotic adjustment. In this process, plants synthesize solutes to adjust their cellular osmotic potential, helping the plant to maintain cellular turgor and protect cellular membranes [1]. A number of compounds such as proline, glutamate, glycine-betaine, fructans, sucrose, oligosaccharides, and inorganic ions such as K^+1^, were documented to be helpful in maintaining the osmotic equilibrium during drought or EM [10].

Extensive research efforts have been carried out in order to improve the crop performance under water deficit or EM. Nevertheless, water deficit or EM is still a major limitation to crop growth and productivity. Typically, seed germination and early seedling growth are less tolerant to environmental fluctuations as compared to mature plants. Thus, soil moisture stress during early growth may result in high mortality rates, leading to poor crop performance [11].

Research demonstrated that seeds osmoprimed with polyethylene glycol (PEG) is effective to improve germination, emergence, and seedling establishment of several plants, especially under stress conditions[12–15]. For example, Hur (1991) reported that germination and seedling establishment of Italian ryegrass (*Lolium multiflorum* L.)[16] and sorghum under drought, cold stress, and high salinity were improved following seed osmopriming with PEG. Similar improvements were also noted for rice (*Oryza sativa* L.) under drought [15] after seed osmopriming with PEG. However, the physiological reasons behind the improved germination and seedling establishment after seed osmopriming are unclear. Therefore, comprehensive research is warranted to elucidate the physiological mechanisms of improved germination performance and stress tolerance after osmopriming with PEG. The objectives of this research were to evaluate the effect of sorghum seed osmopriming with PEG on germination and seedling growth, and to study plant physiology following osmopriming with PEG by exploring the dynamics of antioxidants and stress-related osmolytes in sorghum during the seedling growth stage.

## Materials and Methods

### Experiment description

This research was conducted at Liaoning Academy of Agricultural Sciences in Shenyang, China, 2015. Sorghum (cv. Liao waxy No. 3) seeds were primed by soaking in aerated 20% (w/v) PEG 8000 solution for 48 h at 18°C. After priming, seeds were rinsed thoroughly with distilled water and dried to their original weight by placing seeds in a thermostatic chamber at 20°C, according to Chen and Arora [12]. Unprimed seeds were used as a control. Plastic pots (24 cm diameter by 20 cm deep) were filled with 1 kg of sieved, air-dried silty loam soil (0.52% organic carbon; 38.3% sand; 17.8% coarse silt; 31.0% fine and medium silt; 12.9% clay; 0.113% total N; 0.170% total P; 2.229% total K; 74 mg Kg^-1^ available N; 16.0 mg kg^-1^ available P; 143 mg Kg^-1^ available K; and a soil pH of 6.7). Pots were placed in an artificial climate chamber set for 28/21°C day/night with an average irradiance of 187 μmol m^-1^ s^-1^, 50% relative humidity, and 12 h photoperiod.

Treatments were the factorial combination of two seed treatments, including primed and unprimed seeds, and five soil moisture treatments (SMT), including (I) a normal water supply of field capacity with approximately 25% soil moisture content (regarded as unstressed control) (II) 15% soil moisture content (drought), (III) 35% soil moisture content (EM), (IV) 8 d drought stress followed by EM, and (V) 8 d EM followed by drought stress. EM was followed by drought stress by withholding irrigation continuously for 6 d to reduce soil moisture content to 15%. Preliminary experiments showed that 15 and 35% soil moisture content caused an adverse effect on sorghum germination and seedling growth. The soil moisture content was maintained daily and was monitored at 5:00 PM local time using a soil moisture sensor (ML3 ThetaProbe, Delta-T Device Ltd, 130 Low Rd, Cambridge, UK) with ±1% accuracy.

### Germination performance

Two separate experiments were conducted to evaluate germination percentage, germination index, and vigor index. Each pot was seeded with 30 primed or unprimed seeds and received one of the aforementioned SMTs. Each pot represented an experimental unit. There were three replicate pots were used for each treatment and totally thirty pots in a randomized block design. Starting from 4 DAP, three seeds in each pot were examined daily. Means of three observations in each pot were used for statistical analysis. At the end of the 10 d testing period, all germinating seeds were collected. Germination was defined by the presence of a radicle greater than its seed length [17]. Germination index and vigor index were calculated using the following formulas according to Sun et al.[17]:

Germination index = Σ(G_t_/T_t_), where G_t_ is the number of seeds germinated on day t and T_t_ is the number of days.

Vigor index = Germination index × fresh weight of radicle.

### Physiological parameters

Two separate experiments were conducted to investigate the physiological mechanisms of improved germination performance and stress tolerance after osmopriming sorghum seed with PEG. Six primed or unprimed sorghum seeds were planted in each pot. Pots were fertilized immediately after planting by spraying 60 ml Hoagland solution [18]. The aforementioned SMTs were applied immediately after planting. Plants were thinned to three per pot after emergence. Each SMT comprised three replicate pots. Each pot represented an experimental unit, and each of the three seedlings in a pot is a sampling unit. There were a total of 120 pots arranged in a randomized block design. Relative water content (RWC), chlorophyll, root viability, antioxidant system, lipid peroxidation, O^2−^ content, plasma membrane stability, and osmotic adjustment were determined at 12 and 24 DAP.

Relative water content (RWC) of leaves and roots of sorghum were determined using a formula of RWC (%) = [(FW–DW)/(TW–DW)] × 100, where FW, DW, and TW are fresh weight, dry weight, and turgid weight, respectively. DW was determined when sample weight stabilized in an oven at 65°C. TW was measured 24 h after the saturation of plant samples in deionized water at 4°C [19].

Chlorophyll a and b content was determined with the procedure as described by Arnon [20]. Fresh leaves were cut into 0.5 cm fragments and extracted for 24 h using 80% acetone at -10°C. The resulting extract was centrifuged and the absorbance of the supernatant was measured at 645 and 663 nm using a spectrophotometer (UV-2401, Shimadzu Corporation, Japan).

Root viability was determined by measuring the activity of dehydrogenase using the 2,3,5-triphenyl tetrazolium chloride (TTC) reduction method. Fresh root material (0.2 g) was sampled from the root base, middle root, and root tip. Root material was then cleaned with distilled water and incubated in a 10 ml solvent mixture containing 5 ml 0.4% v/v TTC and 5 ml 0.06 mol∙L^-1^ phosphate buffer (pH 7.0) in darkness at 37°C for 3 h. The reaction was terminated by adding 2 ml of 1 mol L^-1^ sulfuric acid in the tubes. Samples were centrifuged and absorbance of supernatant was measured at 485 nm using a spectrophotometer. Root viability was expressed as the quantity of TTC reduced per gram of root dry mass per h (μg g^-1^ h^-1^).

APX in plant leaves was determined according the procedure described by Nakano and Asada[21] by measuring the decrease in absorbance of the oxidized ascorbate at 290 nm. A 1 ml reaction mixture contained 50 mM potassium phosphate buffer (pH 7), 10 μl enzyme extracts, 0.1 mM H_2_O_2_, and 0.5 mM ascorbate was used. The reaction was initiated by adding H_2_O_2_.

SOD activity was determined following the method of Giannopolitis and Ries [22], with minor modifications. Fresh leaf material (0.2 g) was used for measuring SOD activity. A 3 ml reaction solution containing 50 μM nitroblue tetrazolium (NBT), 1.3 μM riboflavin, 13 mM methionine, 75 nM EDTA, 50 mM phosphate buffer (pH 7.8), and 30 μl of enzyme extract. The test tubes were irradiated under 15 fluorescent lamps at 78 μmol m^-1^ s^-1^ for 15 min. The absorbance of the irradiated solution was 560 nm with a spectrophotometer. The amount of enzyme required to cause 50% photoreduction of NBT was regarded as one unit of SOD activity.

CAT and POD activities in plant leaves were determined using the method developed by Bradford [23], with slight modifications. A 3 ml CAT reaction solution containing 100 μl enzyme extract, 5.9 mM H_2_O_2_, and 50 mM phosphate buffer (pH 7.0) was used. The biochemical reaction was initiated by adding the enzyme extract. Changes in absorbance of the reaction solution at 240 nm were read for every 20 s to determine CAT activity. Similarly, a 3 ml POD reaction solution contained 20 mM guaiacol, 50 mM phosphate buffer (pH 5), and 40 mM H_2_O_2_. Changes in absorbance were read at 470 nm for every 20 s to determine POD activity. One unit of CAT or POD activity was defined as an absorbance change of 0.01 units per min.

Malondialdehyde (MDA) content was measured according to a modification of the method used by Noreen et al.[24]. Fresh leaf (1.0 g) was homogenized in 3 ml 1.0 w/v trichloroacetic acid (TCA) at 4°C and centrifuged at 12000 g for 10 min. A 0.5 ml of supernatant was transferred to 3 ml 0.5 v/v thiobarbituric acid (TBA) in 20% TCA. The resulting mixture was incubated in boiling water for 50 min. After cooling in an ice water bath, the mixture was centrifuged at 12000 g for 15 min. The absorbance of supernatant was read at 532 and 600 nm with a spectrophotometer.

The determination of O^2−^ content was conducted by using a modification of the procedure described by Doke [25]. The O^2−^ content was determined based on its ability to reduce NBT. Fresh leaf tissues (0.5 g) were excised and immersed in 10 mM potassium phosphate buffer (pH 7.8), containing 0.05% nitro blue tetrazolium and 10 mM NaN_3_. The sample was incubated for 1 h at room temperature. Following incubation, 2 ml of this reaction solution was heated at 85°C for 15 min and cooled in an ice bath. Optical density of solution was determined at 560 nm for 15 min using a spectrophotometer. The O^2−^ content was expressed as the increase in absorbance per unit dry weight.

Relative electrolyte leakage was measured to determine the membrane permeability, according to the method by Blum and Ebercon [26]. Sorghum leaf materials were sampled and excised to 5 mm segments. Leaf tissues were rinsed with distilled water and immersed in a test tube containing 6 ml distilled water for 12 h at 18°C. The relative electrolyte leakage of solution was measured (E_1_) using a conductivity meter (Model DDS, Shanghai Leici Instrument Inc., Shanghai, China). Samples were subsequently autoclaved for 10 min at 120°C. After cooling to 25°C, the second relative electrolyte leakage was measured (E_2_). The conductivity of deionized water was also measured (E_0_). The relative electrolyte leakage was determined with the formula of electrolyte leakage (%) = (E_1_ –E_0_)/(E_2_ –E_0_) × 100.

The free amino acid pool in plant leaves was determined according to a minor modification of the procedure used by Moore and Stein[27]. Plant leaf material (0.5 g) was sampled and homogenized with 10 ml of 80% boiling ethanol. The homogenate was centrifuged at 5000 g for 10 min. This extraction was repeated four times and the supernatants were combined and transferred to new tubes. The ethanol extract was evaporated in a fume hood and the residue was dissolved in 5 ml 0.2 M citrate buffer (pH 5.0). A 2 ml aliquot of the sample was mixed with 1 ml of ninhydrin reagent in methyl cellosolve and 0.2 M acetate buffer. The samples were boiled for 20 min and cooled at room temperature. Absorbance was then read at 570 nm with a spectrophotometer.

Proline content in leaf samples was determined following the method of Bates et al. [28]. Fresh leaf material (0.5 g) was homogenized in 10 ml 3% sulphosalicylic acid and centrifuged at 1200 g for 10 min. A 2 ml supernatant was mixed with 2 ml acid ninhydrin reagent and 2 ml glacial acetic acid. The sample was subsequently incubated at 100°C for 60 min. The sample materials were cooled in an ice bath prior to adding 4 ml toluene to each sample. The toluene layer was read at 520 nm with a spectrophotometer.

Soluble and reducing sugars in plant leaves were determined following a modification of the methods used by Dubois et al.[29] and Van Handel [30], with minor modifications. Plant leaves (0.25 g) were placed in a boiling water bath for 1 h. Total soluble sugar content was subsequently analyzed with the phenol-sulfuric method after hydrolysis of starch using perchloric acid. Sucrose content was determined using the anthrone method. Reducing sugar content was calculated as the difference between total soluble sugar and sucrose.

### Experimental design and statistical analysis

Experimental design was a randomized complete block and was repeated in time twice. All measurements were carried out with three replicates. Data were subjected to ANOVA with SAS (100 SAS Campus Dr., Cary, NC 27513) and significance of main effects was determined at the 0.05 probability level. Treatment means were separated using Fisher’s Protected LSD test at P ≤ 0.05. Experiment by treatment interactions were not detected and thus, results were pooled over experiments for analysis.

## Results

### Germination performance

Results indicated that ST, SMT, and their interactions had statistically significant effects on germination percentage, germination index, and vigor index (Table 1). Unprimed and primed seeds germinated under normal soil moisture conditions exhibited greater germination performance than those germinated under suboptimal soil moisture environments. In general, primed seeds had a better germination performance than unprimed seeds in all germination environments, which was indicated by a greater germination percentage, germination index, and vigor index. Moreover, drought stress had a greater adverse effect on germination performance as compared to other SMTs. Seed priming, however, significantly improved germination performance under drought and SMTs. Germination percentages of unprimed seeds under drought stress were 41.3 and 54.4% at 4 and 10 DAP, respectively. In comparison, germination percentages of primed seeds under drought stress were 59.0 and 73.7% at 4 and 10 DAP, respectively.

**Table 1:** Effect of seed priming with PEG and soil moisture stress on sorghum germination.^a^

ST ^b^	SMT	Germination percentage (%)		Germination index	Vigor index
		4 DAP	10 DAP		
Unprimed	Unstressed control	84.9	90.5	25.9	0.81
	Drought	41.3	54.4	10.2	0.26
	EM	59.3	66.8	13.4	0.52
	Drought–EM	46.5	72.5	15.6	0.69
	EM-drought	55.6	73.3	12.3	0.38
Primed	Unstressed control	89.8	96.5	28.6	0.92
	Drought	59.0	73.7	16.4	0.64
	EM	69.4	70.4	15.3	0.59
	Drought–EM	71.7	85.4	20.2	0.85
	EM-drought	70.4	82.3	15.7	0.72
	LSD_0.05_	3.4	5.8	0.9	0.04
ST F-value		17.8*	97.0*	352.8*	606.92*
SMT F-value		13.0*	97.8*	677.5*	322.48*
ST × SMT F-value		33.7*	7.3*	17.7*	53.01*

Numbers represent F-values at 0.05 probability level

* Significant at 0.05 probability level

^a^Sorghum seeds were primed with 20% (w/v) PEG 8000 at 18°C for 24 h. The unprimed and primed sorghum seeds were germinated under unstressed soil moisture environment (Unstressed control), drought, EM, 8 d drought stress followed by EM, and 8 d EM followed by drought stress, respectively.

^b^Abbreviations: DAP, days after planting; EM, excessive soil moisture; LSD, least significant difference; PEG, polyethylene glycol; ST, seed treatment; SMT, soil moisture treatment

### Relative water content

There was significant decline in plant leaf, root, and stem RWC under adverse SMTs as compared to unstressed controls (Table 2). Seed priming, however, considerably enhanced plant leaf, root, and stem RWC across all soil moisture conditions. According to the variance analysis, the effects of ST and SMT were significant for plant leaf, root, and stem RWC at 12 and 24 DAP, while a significant interaction of ST and SMT was only detected for plant stem RWC at 12 DAP. Moreover, a greater decline in plant leaf, root, and stem RWC was noted under drought than other SMTs. At 12 and 24 DAP, plants originating from unprimed seeds suffered a greater decline in RWC when under drought stress than those under other SMTs. The leaf, root, and stem RWC at 24 DAP of plants grown under drought stress conditions that originated from unprimed seeds were 75.3, 73.3, and 72.3%, respectively. However, seed priming greatly increased mentioned parameters to 83.3, 77.3, and 76.2%, respectively. At 24 DAP, plant leaf, root, and stem RWC from unprimed seeds under EM condition were 79.6, 77.3, and 73.3%, respectively. On the contrary, plant leaf, root, and stem RWC from primed seeds under EM condition were 85.9, 82.2, and 79.2%, respectively. Overall, results showed that seed priming significantly improved plant leaf, root, and stem RWC across all SMTs.

**Table 2:** Effect of seed priming with PEG and soil moisture stress on leaf, root, and stem RWC in sorghum seedlings.^a^

		RWC (%)					
		12 DAP			24 DAP		
ST ^b^	SMT	Leaf	Root	Stem	Leaf	Root	Stem
Unprimed	Unstressed control	87.6	84.6	81.1	86.6	82.3	79.3
	Drought	73.1	69.1	68.3	75.3	73.3	72.3
	EM	78.1	75.1	74.6	79.6	77.3	73.3
	Drought -EM	81.3	77.3	76.6	82.3	79.1	77.6
	EM-drought	80.2	76.2	75.5	80.6	78.1	75.5
Primed	Unstressed control	89.2	87.2	83.2	88.5	84.2	82.1
	Drought	81.6	79.6	79.5	83.3	77.3	76.2
	EM	84.2	81.2	78.8	85.9	82.2	79.2
	Drought -EM	85.1	80.1	80.3	86.2	82.3	80.2
	EM-drought	86.2	82.2	79.4	86.0	80.5	80.3
	LSD_0.05_	4.7	4.4	5.0	4.0	4.5	3.7
ST F-value		37.3*	43.1*	32.7*	35.1*	16.2*	38.5*
SMT F-value		16.0*	18.0*	9.2*	9.4*	9.8*	11.7*
ST× SMT F-value		1.6 ns	2.6 ns	3.2*	1.1 ns	0.4 ns	0.9 ns

Numbers represent F-values at 0.05 probability level

*, ns Significant and non-significant at 0.05 probability level

^a^Seeds were primed with 20% (w/v) PEG 8000 at 18°C for 24 h. The unprimed and primed sorghum seeds were germinated under unstressed soil moisture environment (Unstressed control), drought, EM, 8 d drought stress followed by EM, and 8 d EM followed by drought stress, respectively.

^b^Abbreviations: DAP, days after planting; EM, excessive soil moisture; LSD, least significant difference; PEG, polyethylene glycol; RWC, relative water content; ST, seed treatment; SMT, soil moisture treatment

### Chlorophyll content

Variance analysis showed that ST, SMT, and their interactions had significant effects on chlorophyll a and b (Table 3). The highest content of chlorophyll a and b was found in plants originated from primed seeds and grown under unstressed SMTs. Results showed that seed priming largely prevented seedlings grown under adverse soil moisture conditions from chlorophyll loss. Chlorophyll a and b were somewhat restored at 24 DAP in plants grown under adverse SMTs but chlorophyll content in plants originating from unprimed seeds were less than those of plants originating from primed seeds.

**Table 3:** Effect of seed priming with PEG and soil moisture stress on chlorophyll content in sorghum seedlings.^a^

		Chlorophyll content (mg g^-1^ FW)			
		12 DAP		24 DAP	
ST^b^	SMT	Chlorophyll a	Chlorophyll b	Chlorophyll a	Chlorophyll b
Unprimed	Unstressed control	1.17	0.57	1.20	0.59
	Drought	0.81	0.47	0.88	0.51
	EM	0.93	0.45	0.97	0.49
	Drought -EM	0.97	0.41	1.03	0.48
	EM-drought	0.84	0.53	0.88	0.58
Primed	Unstressed control	1.26	0.59	1.31	0.58
	Drought	1.08	0.49	1.13	0.55
	EM	1.14	0.52	1.15	0.57
	Drought -EM	1.19	0.55	1.22	0.56
	EM-drought	0.98	0.61	0.96	0.75
	LSD_0.05_	0.06	0.02	0.08	0.05
ST F-value		235.54*	148.86*	181.06*	167.86*
SMT F-value		80.50*	68.83*	89.67*	94.54*
ST× SMT F-value		6.46*	15.15*	5.90*	27.52*

Numbers represent F-values at 0.05 probability level

* Significant at 0.05 probability level

^a^Seeds were primed with 20% (w/v) PEG 8000 at 18°C for 24 h. The unprimed and primed sorghum seeds were germinated under unstressed soil moisture environment (Unstressed control), drought, EM, 8 d drought stress followed by EM, and 8 d EM followed by drought stress, respectively.

^b^Abbreviations: DAP, days after planting; EM, excessive soil moisture; LSD, least significant difference; PEG, polyethylene glycol; ST, seed treatment; SMT, soil moisture treatment

### Root viability

Results showed that root tips had greater root viability than middle and base portions of roots (Table 4). Results of variance analysis indicated that ST, SMT, and their interactions had significant effects on the viability of root bases, middle roots, and root tips at 24 DAP. Root viability benefited from the priming treatment. Under all SMTs, seed priming significantly increased root viability.

**Table 4:** Effect of seed priming with PEG and soil moisture stress on sorghum root viability.^a^

		Root viability (μg TTC g^-1^ FW h^-1^)					
		12 DAP			24 DAP		
ST^b^	SMT	Tip	Middle	Base	Tip	Middle	Base
Unprimed	Unstressed control	124.69	78.32	45.32	133.35	89.35	46.29
	Drought	86.42	49.14	22.94	119.86	68.57	26.85
	EM	98.24	53.34	19.77	79.15	55.24	23.37
	Drought -EM	79.85	62.85	30.28	103.65	77.22	36.54
	EM-drought	106.37	66.69	32.35	114.13	69.85	38.87
Primed	Unstressed control	133.67	85.47	52.31	139.29	98.24	54.29
	Drought	113.68	68.74	38.29	126.36	79.67	42.31
	EM	103.64	56.38	23.37	83.64	61.33	44.36
	Drought -EM	88.74	48.86	30.37	117.64	80.56	46.24
	EM-drought	114.17	73.54	41.46	118.28	73.39	48.71
	LSD_0.05_	0.63	0.54	0.42	0.78	0.50	0.18
ST F value		8.71*	0.70 ns	7.40 ns	15.21*	18.96*	28.35*
SMT F value		13.85*	12.19*	3.64 ns	102.63*	57.58*	6.44*
ST× SMT F value		973.55*	1098.04*	1447.75*	153.76*	263.67*	1150.40*

Numbers represent F-values at 0.05 probability level

*, ns Significant and non-significant at 0.05 probability level

^a^Seeds were primed with 20% (w/v) PEG 8000 at 18°C for 24 h. The unprimed and primed sorghum seeds were germinated under unstressed soil moisture environment (Unstressed control), drought, EM, 8 d drought stress followed by EM, and 8 d EM followed by drought stress, respectively.

^b^Abbreviations: DAP, days after planting; EM, excessive soil moisture; LSD, least significant difference; PEG, polyethylene glycol; ST, seed treatment; SMT, soil moisture treatment

### Antioxidant enzymes

**Fig 1:**
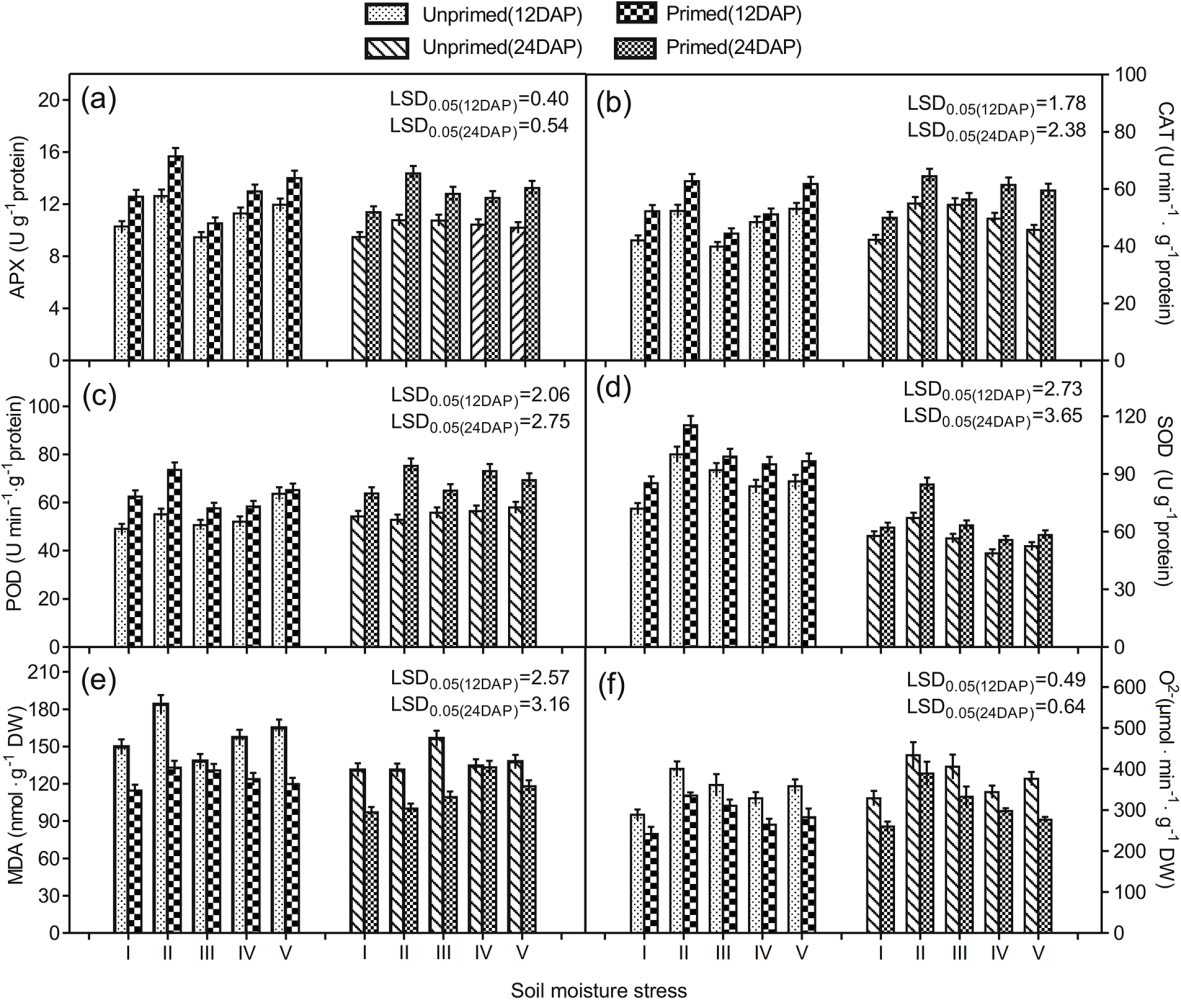
Effect of seed priming with PEG and soil moisture stress on sorghum APX, CAT, POD, SOD, MDA, and O^2−^ activities. (a) Ascorbate peroxidase (APX), (b) catalase (CAT), (c) malondialdehyde (MDA), (d) hydrogen peroxide (H_2_O_2_), (e) peroxidase (POD), and (f) superoxide dismutase (SOD) activities in sorghum seedlings. Unprimed or primed sorghum seeds were germinated under (I) unstressed soil moisture environment (Unstressed control), (II) drought, (III) excessive soil moisture stress (EM), (IV) 8 d drought stress followed by EM, and (V) 8 d EM followed by drought stress. Measurements were conducted at 12 and 24 days after planting (DAP). Data presented are means ± standard errors.

Activities of selected ROS scavenging enzymes, including APX, CAT, POD, and SOD, were determined in plant leaves at 12 and 24 DAP. Overall, APX and CAT activity was strongly affected by seed priming and SMT as indicated by the significant effects of ST, SMT, DAP, and ST by SMT and SMT by DAP interactions (Table 5). Variance analysis indicated that ST, SMT, DAP, and several of their interactions had statistically significant effects on POD activity. Similarly, SOD activity was significantly affected by seed priming and SMT as indicated by the significant effects of ST, SMT, DAP, and the SMT by DAP interaction. Overall, priming substantially increased the activities of APX, CAT, POD, and SOD across all SMTs (Fig 1).

**Table 5:** Results of variance analysis of ST, SMT, DAP, and their interactions for APX, CAT, POD, SOD, MDA, and O^2−^ activities.^a^

Independent variable	Dependent variable					
	APX	CAT	POD	SOD	MDA	O^2−^
ST	326.60*	214.04 *	322.26*	142.30 *	556.29 *	654.37*
SMT	53.60*	59.78 *	22.60*	96.08*	15.28*	65.23*
DAP	18.85 *	30.90*	30.63*	146.79*	156.37*	23.31*
ST×SMT	5.89 *	6.21*	14.18*	4.07 ns	10.56*	13.59*
ST×DAP	3.83 ns	2.07 ns	12.16*	3.95 ns	9.82*	2.35 ns
SMT×DAP	22.55 *	31.08 *	9.31*	16.92*	25.57*	2.81 ns
ST×SMT×DAP	1.04 ns	4.52 ns	4.21 ns	1.39 ns	18.63 *	4.65 *

Numbers represent F-values at 0.05 probability level

*, ns Significant and non-significant at 0.05 probability level

^a^Abbreviations: APX, ascorbate peroxidase; CAT, catalase; DAP, days after planting; O^2−^, superoxide radicals; MDA, malondialdehyde; POD, peroxidase; SOD, superoxide dismutase; ST, seed treatment; SMT, soil moisture treatment

### Lipid peroxidation

MDA levels in plant leaves were determined to evaluate lipid peroxidation. Results showed that ST, SMT, and DAP, and their interactions had significant effects on MDA activity (Table 5). At 12 and 24 DAP, MDA had accumulated at lower levels in plants originating from primed seeds compared to plants originating unprimed controls (Fig 1). At 24 DAP, Overall, priming remarkably reduced the extent of MDA accumulation in plants treated with adverse SMTs.

### Superoxide radicals

Results showed that ST, SMT, DAP, and ST by SMT and ST by SMT by DAP interactions had significant effects on O^2−^ accumulation in plant leaves (Table 5). At 12 and 24 DAP, significant decreases in O^2−^ content were evident in plants originating from primed seeds compared to unprimed controls, regardless of SMT (Fig 1). At 24 DAP, seed priming resulted in a reduction in O^2−^ content in plant leaves grown under unstressed environment, drought, EM, drought followed by EM, and EM followed by drought, by 18, 16, 19, 17, and 28%, respectively. Overall, our data showed that the priming treatment significantly reduced O^2−^ accumulation in sorghum seedlings under various suboptimal soil moisture environments at 12 and 24 DAP.

### Membrane permeability

**Fig 2:**
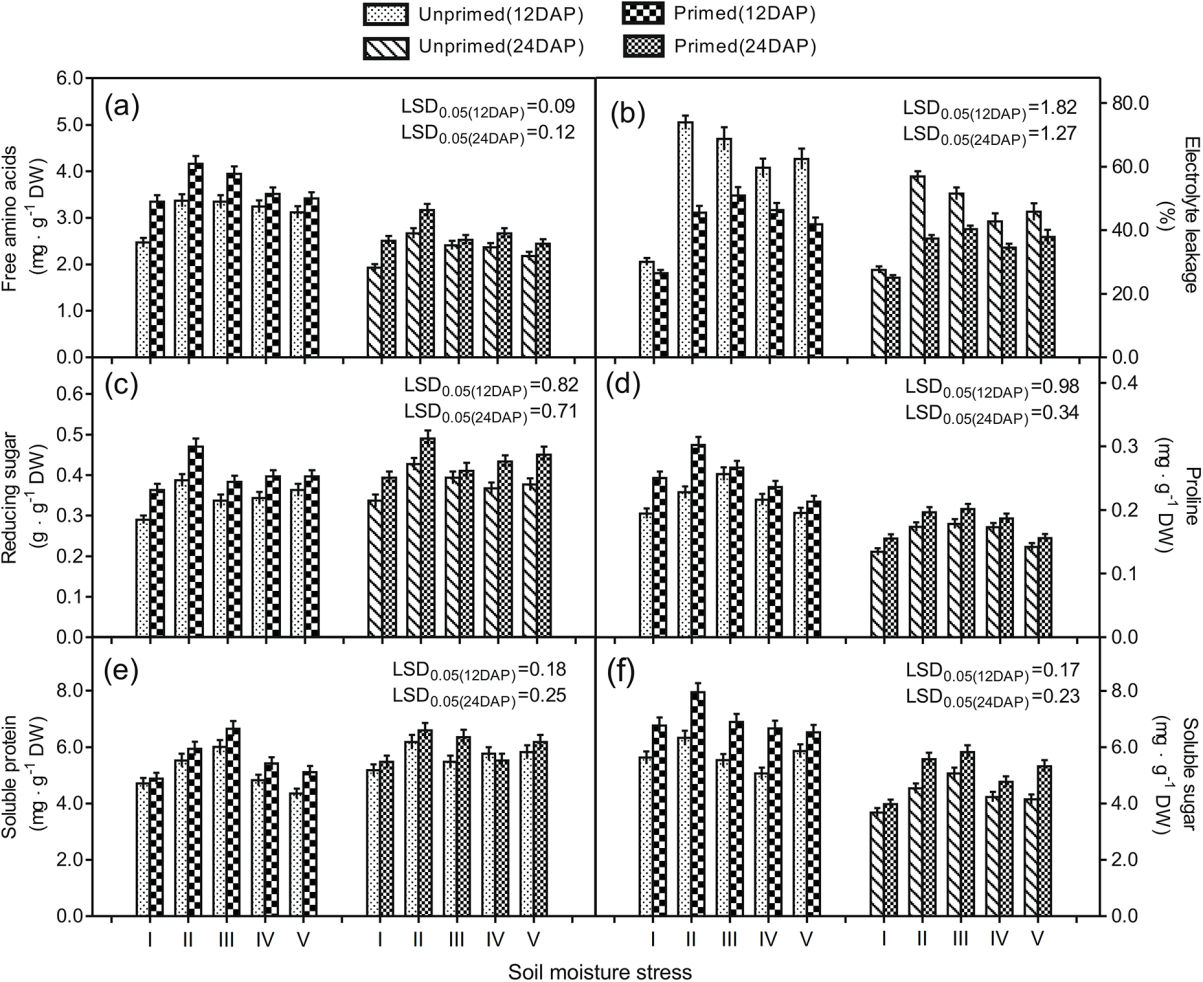
Effect of seed priming with PEG and soil moisture stress on sorghum free amino acid pool, proline, plasma membrane permeability, reducing sugar, soluble sugar, and soluble protein activities. (a) Free amino acid, (b) electrolyte leakage, (c) reducing sugar, (d) proline, (e) soluble protein, and (f) soluble sugar in sorghum seedlings. Unprimed or primed sorghum seeds were germinated under (I) unstressed soil moisture environment (Unstressed control), (II) drought, (III) excessive soil moisture stress (EM), (IV) 8 d drought stress followed by EM, and (V) 8 d EM followed by drought stress. Measurements were conducted at 12 and 24 days after planting (DAP). Data presented are means ± standard error.

The data showed that all independent variables including ST, SMT, DAP, and their interactions had significant effect on membrane permeability except for the effect of the ST by SMT by DAP interaction (Table 6). All adverse SMTs significantly increased membrane permeability at 12 and 24 DAP, as indicated by increased electrolyte leakage (Fig 2). At 24 DAP, electrolyte leakages of plants originated from unprimed seeds grown under drought, EM, drought followed by EM, and EM followed by drought were 59, 53, 45, and 47%, respectively. However, seed priming greatly reduced mentioned parameters to 38, 40, 37, and 38%, respectively. Results suggest that seed priming was effective in decreasing membrane permeability in sorghum seedlings, as indicated by reduced electrolyte leakage.

**Table 6:** Results of variance analysis of ST, SMT, DAP, and their interactions for free amino acid pool, proline, plasma membrane permeability, reducing sugar, soluble sugar, and soluble protein activities.^a^

Independent variable	Dependent variable					
	Electrolyte leakage	Free amino Acid	Proline	Reducing sugar	Soluble sugar	Soluble protein
ST	655.06*	211.15*	156.73*	190.81*	52.64*	295.50*
SMT	334.04*	68.28*	86.30*	57.31*	49.62*	45.68*
DAP	417.83*	825.07*	921.31*	70.95*	74.42*	736.49*
ST×SMT	42.01*	9.13*	9.83*	2.38ns	3.26ns	2.87ns
ST×DAP	43.51*	11.28*	14.52ns	0.17ns	2.22ns	19.02ns
SMT×DAP	19.18*	6.39ns	8.95ns	0.35ns	20.49*	22.62*
ST×SMT×DAP	3.42ns	2.21ns	7.08ns	2.45ns	2.71ns	5.30ns

Numbers represent F-values at 0.05 probability level

*, ns Significant and non-significant at 0.05 probability level

^a^Abbreviations: DAP, days after planting; ST, seed treatment; SMT, soil moisture treatment

### Stress-related osmolytes

Results of variance analysis showed that ST, SMT, DAP, and the ST by SMT interaction had significant effects on the free amino acid pool and proline content of seedlings (Table 6). ST, SMT, and DAP had significant effects on reducing sugar. Additionally, seed priming and SMT had significant effect on soluble protein levels as indicated by significant effects of ST, SMT, DAP, and the SMT by DAP interaction. Overall, results suggest that seed priming enhanced reducing sugar, proline, and soluble sugar content in plant leaves across all SMTs (Fig 2).

## Discussion

Results of this study revealed that seed priming could invigorate sorghum seeds, resulting in greater germination performance under various soil moisture conditions (Table 1). In the present study, earlier and more uniform germination and emergence were noted with primed seeds as indicated by greater germination percentage, germination index, and vigor index. These results are in agreement with previous findings with *Bromus* species, canola (*Brassica napus* L.), rice, and sunflower (*Helianthus annuus* L.) [15, 16, 31]. Seed germination and early seedling establishment are the most critical stages for any crop. Unfavorable soil moisture conditions including drought and EM may severely reduce germination rate and uniformity [11]. Therefore, the benefits of seed priming may be more apparent under unfavorable soil moisture environments compared to more optimal conditions [32].

In the present experiment, the decline of RWC of sorghum seedlings grown under drought and EM conditions agrees with the previous findings. For example, RWC of Jarrah (*Eucalyptus marginata* Donn ex Sm.) [33] and welsh onion (*Allium fistulosum* L.)[34] decreased under EM. RWC of corn (*Zea mays* L.) [35] also decreased under drought stress. Overall, priming treatment resulted in better germination performance and increased RWC in sorghum seedling under various suboptimal soil moisture conditions.

In response to drought or EM, plants exhibit wilting, yellowing, and progressive senescence because of reduced chlorophyll content[36]. The present study suggested that priming treatment improves chlorophyll content (Table 3), and thus inhibits leaf senescence. It was found that under drought or EM conditions, leaf chlorophyll content decreased due to the chloroplasts damage caused by ROS [34, 37]. Furthermore, results showed that root viability of sorghum seedlings is adversely affected by suboptimal soil moisture conditions (Table 4). Root viability indicates the integrity of root cell membrane. Thus, root viability is a valid indicator of root injury and seedling survivability under adverse soil moisture conditions [38]. The declines in leaf chlorophyll content and root viability under adverse soil moisture conditions, and variations among plants originating from unprimed or primed seeds, may be associated with the effects of soil moisture stress on the balance between ROS production and ROS scavenging.

The content of MDA represented the level of lipid peroxidation. MDA is produced when polyunsaturated fatty acids in the cellular membrane suffer oxidative damage through the accumulation of ROS. Plasma membrane stability in plant leaves was significantly reduced as indicated by greater electrolyte leakage under suboptimal soil moisture environments (Fig 2). Increased electrolyte leakage is often noted as a symptom of stress related injury, caused by the accumulation of ROS [1, 24, 26]. In the present study, O^2−^ and MDA contents were increased, while membrane stability was decreased under unfavorable soil moisture conditions (Fig 1), suggesting oxidative damage to the plant tissues. Seed priming, however, significantly alleviated the adverse soil moisture effects, which were evident from the considerably enhanced membrane stability observed as a function of lowered electrolyte leakage (Fig 2) and reduced O^2−^ and MDA contents (Fig 1).

Plants have defense mechanisms to alleviate damage caused by ROS. Antioxidant enzymes including APX, CAT, POD, and SOD are noted to be effective against oxidative damage [1, 8]. These antioxidant enzymes control lipid peroxidation and cell membrane stability by scavenging ROS [1, 8]. Our results showed that the improved cell membrane stability and reduced lipid peroxidation in sorghum seedlings originating from primed seeds were accompanied by increased activities of antioxidant enzymes including APX, CAT, POD, and SOD (Fig 1 and Fig 2). This suggested that seed priming largely improved the enzymatic antioxidant activities in sorghum seedlings, which subsequently improved plant growth during seedling stage under unfavorable SMTs. Our results are in accordance with Zhang et al.[39], who reported that SOD, POD, and CAT activities in perilla mint (*Perilla frutescens* L. Britt) seedlings increased following the seed priming with PEG.

It is well known that proline content in plant leaves is enhanced by several stresses including drought and EM [28, 36, 40, 41]. Our results showed that proline contents were considerably higher in plant leaves under adverse SMTs and were remarkably enhanced by seed priming (Fig 2). The free amino acid pool and soluble protein content were higher under adverse SMTs and remarkably enhanced by seed priming, especially at the earlier seedling stage of 12 DAP (Fig 2). The accumulation in the free amino acid pool under water stress may be due to the hydrolysis of protein from the process of osmotic adjustment [42]. It appears that the increase in free amino acid content under water stress is an adaptive mechanism. Seed priming was shown to be effective in strengthening this physiological adaptation, although the promotive effect tended to be weakened at the end of the day of the experiment. Similar results were reported by Chen and Arora [12], who found that the promotive effect of osmoprimed seeds with PEG on stress tolerance of spinach (*Spinacia oleracea* L.) plants may diminish in relatively mature seedlings.

Seed germination and early seedling establishment predominately depend on the mobilization of soluble sugars from storage seed tissues to various organs like the radical and stem, where they are needed for maintaining osmotic homeostasis and plant growth [43, 44]. Like other cellular constituents, sugar contents are influenced by environmental stress. There are contradictory results in previous reports on the effect of water stress on sugar accumulation. For example, some researchers noted that sugar content decreased [45] or remained constant [46], while others noted sugar content increased under water stress [40]. In the present study, the increased reducing and soluble sugar content in plants under water stress may act as compatible solutes in sorghum seedlings. Seed priming appears to be effective in improving the content of these compatible solutes, thus protecting sorghum seedling growth under suboptimal soil water conditions.

Increased RWC in sorghum seedlings originating from primed seeds is likely due to increased compatible solutes including free amino acid, reducing sugar, proline, soluble sugar, and soluble protein contents. Greater levels of compatible osmolytes may have allowed seedlings to maintain appropriate water potential, thus offsetting the adverse effect of water stress [47].

In this research, we verified that seed priming with PEG promoted sorghum germination and seedling growth under various adverse SMTs. Sorghum seeds primed with PEG had significantly improved chlorophyll content and root viability, and priming helped sorghum seedlings to maintain RWC under adverse soil moisture environments. The priming treatment enhanced the activities of antioxidant enzymes including APX, CAT, POD, and SOD, increased contents of free amino acid, proline, and reducing and soluble sugars, and reduced MDA accumulation and electrolyte leakage in sorghum seedlings grown under suboptimal SMTs, therefore resulting in increased stress tolerance.

## Abbreviations

APX: Ascorbate peroxidase
CAT: Catalase
DAP: Days after planting
EM: Excessive soil moisture
LSD: Least significant difference
MDA: Malondialdehyde
SOD: Superoxide dismutase
PEG: Polyethylene glycol
POD: Peroxidase
RWC: Relative water content
ROS: Reactive oxygen species
SMT: Soil moisture treatment
ST: Seed treatment
TBA: Thiobarbituric acid
TCA: Trichloroacetic acid
TTC: 2,3,5-triphenyl tetrazolium chloride
